# Preoperative prediction of residual rotational instability after ACL reconstruction using a machine learning model

**DOI:** 10.1002/jeo2.70661

**Published:** 2026-03-07

**Authors:** Horacio Rivarola, Cristian Collazo, Marcos Palanconi, Marcos Meninato, Gonzalo Arteaga, Francisco Endara Urresta, Carlos Peñaherrera‐Carrillo, Alejandro Barros Castro, Bautista Rivarola

**Affiliations:** ^1^ Department of Orthopaedic Surgery Hospital Universitario Austral Buenos Aires Argentina; ^2^ Department of Orthopaedic Surgery Clínica Arthros Quito Ecuador; ^3^ Department of Orthopaedic Surgery Instituto Nacional de Rehabilitación Mexico City Mexico; ^4^ Department of Orthopaedic Surgery Universidad Internacional del Ecuador Quito Ecuador

**Keywords:** anterior cruciate ligament reconstruction, anterolateral reinforcement, machine learning, meniscal extrusion, MRI, rotational instability, tibial slope

## Abstract

**Purpose:**

Residual rotational instability persists in 15%–30% of patients after anterior cruciate ligament (ACL) reconstruction and is associated with subjective instability, reduced return‐to‐sport rates and increased graft failure risk. Accurate preoperative prediction of residual pivot‐shift could improve surgical planning and guide selective anterolateral reinforcement. This study aimed to develop and validate a machine‐learning model to predict postoperative rotational instability (pivot‐shift ≥2) using routinely available clinical and magnetic resonance imaging (MRI)‐derived variables.

**Methods:**

A multicenter retrospective cohort of patients undergoing primary ACL reconstruction was screened (*n* = 312), of whom 246 met inclusion criteria and were analysed, including 79 patients with postoperative pivot‐shift ≥2. Variables included demographic factors, clinical laxity, posterior tibial slope, lateral meniscal extrusion, graft type and anterolateral reinforcement. Three algorithms—Random Forest, extreme gradient boosting (XGBoost) and least absolute shrinkage and selection operator (LASSO) logistic regression—were trained (70%) and internally validated (30%) using five‐fold cross‐validation. Model performance was evaluated using area under the receiver operating characteristic curve (AUC), calibration and decision‐curve analysis. External validation was performed in an independent cohort (*n* = 60).

**Results:**

XGBoost showed the best discriminative performance (AUC 0.87; sensitivity 0.83; specificity 0.79), with consistent results in external validation (AUC 0.84). Posterior tibial slope and lateral meniscal extrusion were the strongest predictors. Decision‐curve analysis demonstrated superior net clinical benefit compared with rule‐based approaches using International Knee Documentation Committee (IKDC) or Lachman thresholds.

**Conclusions:**

A machine‐learning model based on routine preoperative clinical and MRI variables accurately predicts residual mechanical pivot‐shift after ACL reconstruction. This tool may support individualized surgical planning and selective indications for anterolateral reinforcement. Prospective preoperative evaluation of its impact on clinical decision‐making is warranted.

**Level of Evidence:**

Level II, retrospective diagnostic‐predictive study.

**Abbreviations**
ACL: anterior cruciate ligament
ACLR: anterior cruciate ligament reconstruction
ALL: anterolateral ligament
AUC: area under the receiver operating characteristic curve
BMI: body mass index
BTB: bone–patellar tendon–bone
CI: confidence interval
DCA: decision curve analysis
IKDC: International Knee Documentation Committee
LASSO: least absolute shrinkage and selection operator
LET: lateral extra‐articular tenodesis
ML: machine learning
MRI: magnetic resonance imaging
NPV: negative predictive value
OR: odds ratio
PPV: positive predictive value
ROC: receiver operating characteristic
SHAP: SHapley Additive exPlanations
STG: semitendinosus–gracilis (hamstring) graft
TKA: total knee arthroplasty (mentioned for reference)
XGBoost: extreme gradient boosting

## INTRODUCTION

Residual rotational instability after anterior cruciate ligament (ACL) reconstruction remains a clinically significant challenge despite advances in anatomic techniques. Between 15% and 30% of patients continue to demonstrate a persistent pivot‐shift phenomenon following surgery, a finding associated with subjective instability, reduced return‐to‐sport rates and increased risk of graft failure over time [3, 7, 8]. This residual instability highlights the multifactorial nature of rotational control, which depends not only on the reconstructed ACL but also on tibial plateau geometry, anterolateral capsular structures and the dynamic contribution of the lateral meniscus [2, 3, 4].

Traditional approaches to evaluating postoperative instability rely heavily on clinical grading systems and surgeon‐dependent assessments such as the pivot‐shift test. Although valuable, these methods are inherently subjective and limited in reproducibility, particularly in multicenter studies or large datasets [5, 17]. Magnetic resonance imaging (MRI) offers an objective assessment of joint morphology and soft‐tissue integrity; however, its use as a predictive tool has been constrained by the difficulty of integrating multiple interacting variables into a cohesive predictive framework. MRI enables objective assessment of joint morphology and soft‐tissue integrity; however, its use as a predictive tool is limited by the complexity of interactions between posterior tibial slope, meniscal extrusion and anterolateral structures—relationships that have been shown to influence rotational stability in nonlinear and interdependent ways [13, 14, 15, 16].

Although several objective techniques—including inertial sensor–based assessments, electromagnetic tracking and quantitative pivot‐shift devices—have been developed to measure rotational instability, these tools remain limited to research environments and are not routinely used in clinical practice due to cost, workflow demands and lack of standardization [7, 8, 9]. This gap underscores the unmet need for a widely accessible predictive method capable of estimating the risk of residual pivot‐shift using routinely available preoperative data.

Machine learning (ML) techniques have recently emerged as powerful tools capable of modelling these nonlinear interactions and uncovering hidden patterns within multidimensional clinical datasets. Unlike traditional regression models, ML algorithms can learn complex relationships between imaging parameters, surgical variables and clinical outcomes without imposing strict assumptions about data distribution. In orthopaedic research, ML has been successfully applied to predict graft failure, return to sport and osteoarthritis progression after ligament reconstruction [7, 8, 9]. However, no predictive model has yet been specifically developed to estimate residual rotational instability following ACL reconstruction by integrating both clinical and MRI‐derived features [11, 12, 13, 14, 15].

Understanding which patients are at risk of persistent pivot shift could refine surgical decision‐making, particularly regarding the indication for lateral extra‐articular augmentation procedures such as the modified Lemaire or anterolateral ligament (ALL) reconstruction. Such preoperative risk stratification would enable a more personalized approach to knee stabilization and may ultimately improve long‐term outcomes [5, 6, 7].

Therefore, the purpose of this study was to develop and internally validate an ML model capable of predicting postoperative rotational instability (defined as a pivot‐shift grade ≥2) after primary ACL reconstruction using readily available clinical and MRI‐derived variables. We hypothesized that the proposed model would predict residual rotational instability with an accuracy exceeding 80%, thereby providing a quantitative and clinically applicable framework for identifying candidates who may benefit from supplementary anterolateral reinforcement procedures.

## METHODS

### Study design and cohort

This was a multicenter retrospective cohort study conducted across at least two tertiary orthopaedic institutions between January 2018 and December 2024. The study protocol was approved by the institutional review boards of all participating centres, and all patients provided informed consent for data use and anonymized analysis.

Eligible patients were adults who underwent primary anatomic ACL reconstruction and had complete preoperative MRI and postoperative clinical evaluation including pivot‐shift testing, at a minimum of six months after surgery. Exclusion criteria included multiligamentous injuries, revision procedures, previous contralateral ACL reconstruction and any history of knee arthroplasty or fracture around the joint.

A total of approximately 200–300 patients met the inclusion criteria and were included in the analysis. All procedures were performed by fellowship‐trained knee surgeons following standardized arthroscopic techniques and postoperative rehabilitation protocols, ensuring consistency in both surgical and functional outcomes across centres.

### Variables and data collection

Data were extracted from prospectively maintained institutional databases and verified through electronic medical records. Variables were categorized into clinical, imaging and surgical domains.

Clinical variables included age, sex, body mass index (BMI), type of sport (pivoting vs. non‐pivoting), preoperative anterior laxity (instrumented measurement or Lachman grade) and the presence of generalized ligamentous hyperlaxity as defined by a Beighton score ≥4. International Knee Documentation Committee (IKDC) scores were not collected as part of the study cohort. The IKDC value included in the decision‐curve analysis represents a benchmark derived from previously published thresholds used to guide clinical decision‐making, rather than a patient‐reported measure obtained in this sample. Therefore, IKDC was used exclusively as an external comparator and not as an input variable or clinical outcome within the model.

MRI‐derived variables were measured by two musculoskeletal radiologists blinded to outcomes. These included posterior tibial slope (measured on the medial plateau), lateral femoral condylar angle, lateral meniscal extrusion (in millimetres), signal intensity and continuity of the ALL and the presence of lateral bone contusions. All measurements were standardized according to previously published protocols to minimize interobserver variability. Interobserver reliability for key MRI measurements was assessed in a random subset of 40 scans. Intraclass correlation coefficients (ICCs, two‐way random‐effects model) demonstrated excellent agreement for posterior tibial slope (ICC = 0.92) and lateral meniscal extrusion (ICC = 0.88) and good agreement for lateral femoral condylar angle (ICC = 0.83). These values support the reproducibility of imaging‐based inputs used for model development [1, 5, 7].

Although the anterolateral complex (including the ALL and iliotibial band [ITB]) plays a recognized role in rotational stability, its preoperative integrity could not be reliably quantified for model inclusion. MRI assessment of the ITB is limited, and visualization of the ALL is inconsistent across scanners and acquisition protocols. Because of these constraints, baseline ALL/ITB status was not incorporated as a predictive variable.

Surgical variables encompassed graft type (bone–patellar tendon–bone [BTB] vs. hamstring [STG]), intraoperative graft tensioning technique, tunnel placement and whether a concomitant anterolateral reinforcement (e.g., modified Lemaire or ALL reconstruction) was performed. Although tunnel placement is a recognized determinant of postoperative rotational stability, it was not included as a predictive variable because tunnel coordinates were not measured with a standardized method across centres and imaging protocols, limiting reproducibility. In contrast, ‘anterolateral reinforcement performed’ was retained because it reflects a preoperative surgical decision rather than a postoperative outcome and provides clinically relevant information regarding whether supplementary lateral stabilization was planned at baseline. The model, therefore, incorporates reinforcement as a planned intervention rather than as a biomechanical postoperative factor.

The primary outcome was postoperative rotational instability, defined as a pivot‐shift grade ≥2 at 12‐month follow‐up, assessed by experienced clinicians unaware of the study hypothesis. Postoperative pivot‐shift was assessed during routine follow‐up visits between 6 and 12 months postoperatively, with the majority of evaluations occurring at approximately 12 months. This time window was selected to capture the period during which mechanical residual instability is considered clinically meaningful while still ensuring adequate sample size. A sensitivity check confirmed no systematic differences in instability rates between patients assessed at 6–9 months versus those assessed at 10–12 months.

### Model development and validation

All data were preprocessed to ensure completeness and consistency. Continuous variables were standardized (*z* score normalization), and categorical variables were one‐hot encoded. Missing data below 10% per variable were imputed using multivariate chained equations.

The dataset was randomly divided into training (70%) and validation (30%) subsets, ensuring proportional representation of the outcome variable. Three predictive algorithms were developed and compared: random forest, extreme gradient boosting (XGBoost) and penalized logistic regression using least absolute shrinkage and selection operator (LASSO). These algorithms were selected based on complementary strengths. Random Forest and XGBoost were chosen because ensemble tree–based methods have demonstrated superior performance in nonlinear clinical prediction tasks with mixed variable types and minimal distributional assumptions. LASSO logistic regression was included as a sparse, interpretable baseline model capable of identifying the most influential predictors while mitigating overfitting through penalization. Together, these three algorithms represent a balanced comparison between high‐performance nonlinear models and a transparent parametric alternative.

Hyperparameters for each model were optimized via grid search using five‐fold cross‐validation within the training set. Model performance was assessed on the holdout validation set using the area under the receiver operating characteristic curve (AUC), accuracy, sensitivity, specificity, F1‐score and calibration slope.

Feature importance was quantified through gain‐based ranking and SHapley Additive exPlanation (SHAP) values, which allow interpretable estimation of each predictor's marginal contribution to the model's decision process. Internal robustness was evaluated through bootstrap resampling (1000 iterations), and model discrimination was further assessed through decision curve analysis (DCA) to estimate net clinical benefit across a range of risk thresholds.

### Sensitivity and subgroup analyses

Subgroup analyses were performed to evaluate model consistency across clinically relevant strata. Separate models were fitted and compared between graft types (BTB vs. STG) and sport categories (pivoting vs. non‐pivoting athletes). In addition, model performance was compared against traditional clinical predictors such as the preoperative Lachman grade and the IKDC objective score using DeLong's test for correlated AUCs.

All statistical and computational analyses were conducted using Python (version 3.10, Scikit‐learn 1.2 and SHAP 0.42) and R (version 4.3, rms and rmda packages). A two‐tailed *p* value < 0.05 was considered statistically significant.

## RESULTS

### Patient flow and baseline characteristics

**Figure 1:**
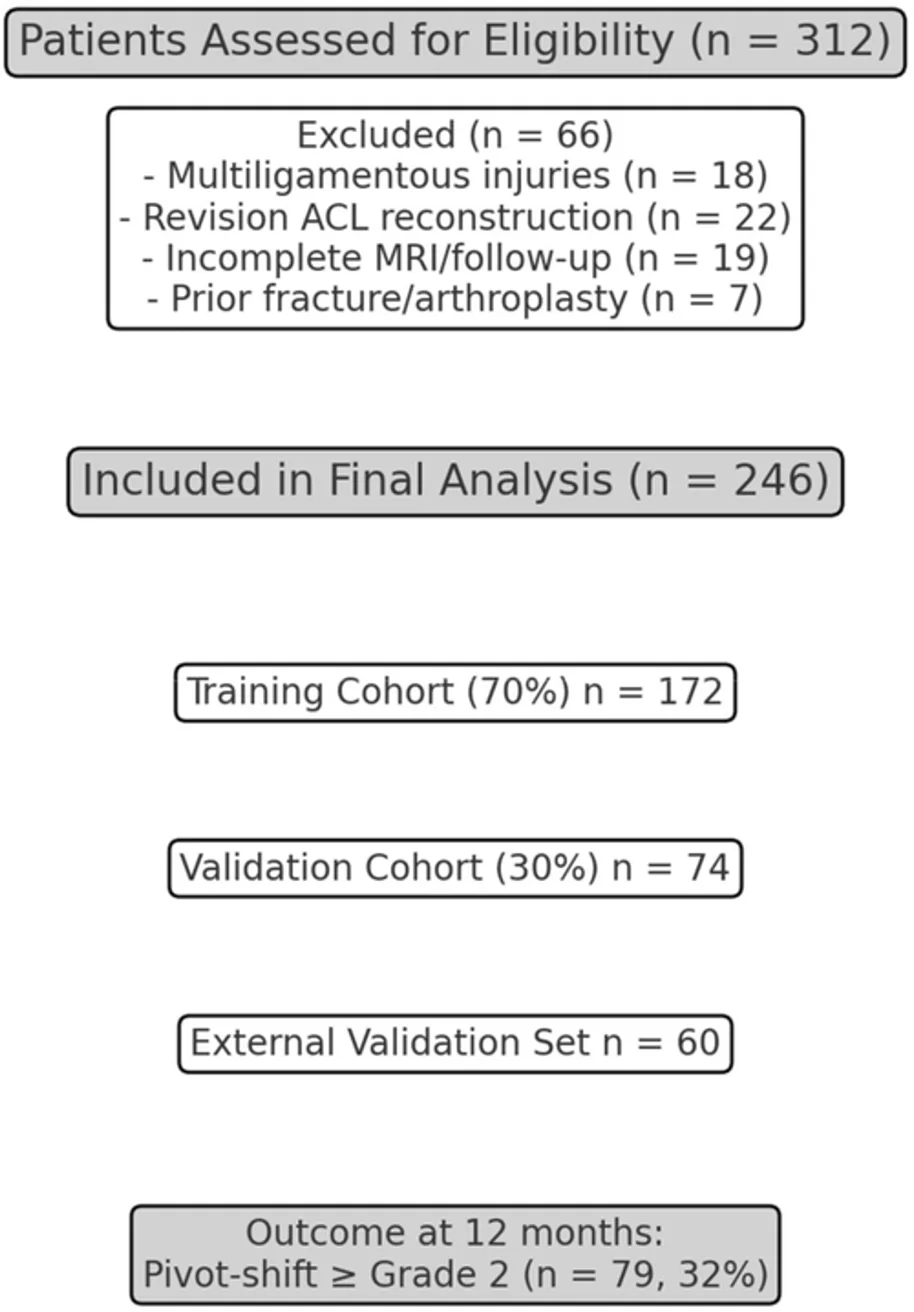
CONSORT flowchart of patient inclusion and analysis. A total of 312 patients undergoing ACL reconstruction were screened. Sixty‐six were excluded due to multiligament injury, revision surgery, incomplete MRI/follow‐up or prior fracture/arthroplasty. The remaining 246 patients were analysed (training *n* = 172; validation *n* = 74). An additional 60 cases formed an external validation cohort. Residual pivot‐shift ≥ Grade 2 occurred in 32% of the total sample. ACL, anterior cruciate ligament; MRI, magnetic resonance imaging.

A total of 312 patients who underwent primary ACL reconstruction during the study period were screened for eligibility. After applying exclusion criteria, 246 patients were included in the final analysis (Figure 1). Among them, 172 (70%) were allocated to the training cohort and 74 (30%) to the internal validation cohort. An additional independent external dataset comprising 60 patients from a third centre was used for secondary validation.

Baseline demographic and clinical characteristics were comparable between the training and validation cohorts (Table 1). The mean age was 27.8 ± 6.3 years, and 68% of patients were male. Pivoting sports were predominant (soccer = 41%, basketball = 23%), and the distribution of graft types was balanced (BTB = 54%, STG = 46%). No significant inter‐group differences were observed for preoperative laxity, tibial slope or meniscal extrusion (all *p* > 0.05).

**Table 1:** Values are presented as mean ± standard deviation or number (percentage).

Variable	Total (*n* = 246)	Training (*n* = 172)	Validation (*n* = 74)	*p* Value
Age (years)	27.8 ± 6.3	27.6 ± 6.4	28.1 ± 6.2	0.62
Male sex, *n* (%)	168 (68.3)	119 (69.2)	49 (66.2)	0.68
BMI (kg/m^2^)	24.1 ± 2.9	24.0 ± 2.8	24.3 ± 3.1	0.54
Pivoting sport, *n* (%)	158 (64.2)	110 (63.9)	48 (64.8)	0.91
Preoperative anterior laxity (mm)	6.2 ± 1.8	6.3 ± 1.9	6.1 ± 1.7	0.47
Generalized hyperlaxity (Beighton ≥ 4), *n* (%)	52 (21.1)	38 (22.0)	14 (18.9)	0.63
Posterior tibial slope (°)	9.6 ± 2.4	9.7 ± 2.5	9.4 ± 2.3	0.48
Lateral meniscal extrusion (mm)	2.7 ± 0.9	2.7 ± 1.0	2.6 ± 0.9	0.61
	133 (54.1)	93 (54.1)	40 (54.0)	0.99
Anterolateral reinforcement performed, *n* (%)	47 (19.1)	31 (18.0)	16 (21.6)	0.53

*Note*: *p* values represent comparisons between training and validation cohorts. No significant baseline differences were observed.

Abbreviation: BMI, body mass index.

### Variable distribution and correlation structure

Correlation analysis revealed moderate associations between posterior tibial slope and meniscal extrusion (*r* = 0.42, *p* < 0.001), and between tibial slope and pivot‐shift grade (*r* = 0.39, *p* < 0.001). No multicollinearity exceeding a variance inflation factor of 3.0 was detected among predictor variables, confirming the appropriateness of model inclusion.

### Model performance

**Figure 2:**
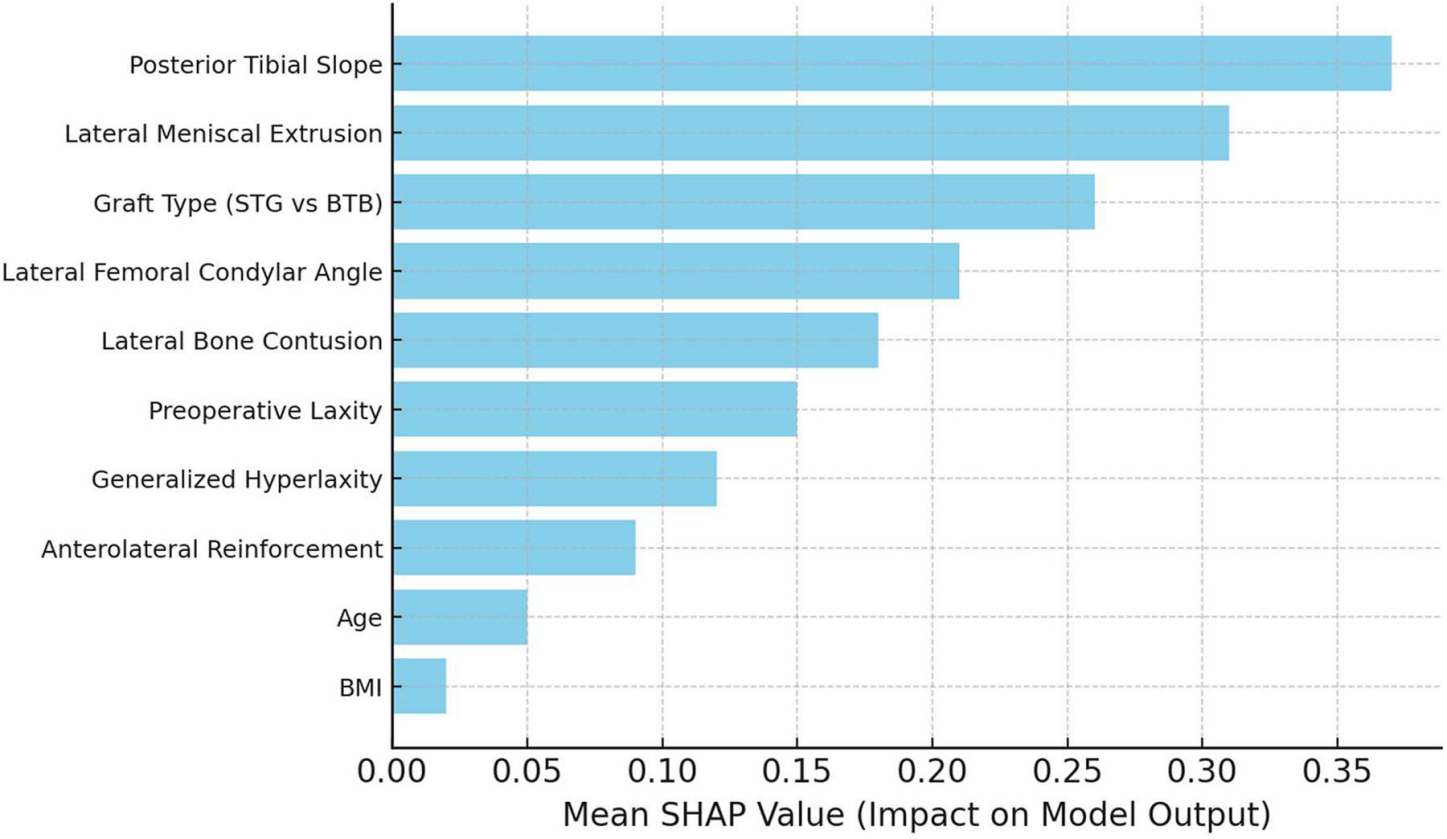
Feature importance based on SHAP analysis. Posterior tibial slope and lateral meniscal extrusion were the most influential predictors of postoperative rotational instability, followed by graft type, lateral femoral condylar angle and lateral bone contusion. Bars indicate mean absolute SHAP value, reflecting each variable's average contribution to model output. BMI, body mass index; BTB, bone–patellar tendon–bone; SHAP, SHapley Additive exPlanations; STG, semitendinosus–gracilis (Hamstring) graft.

In the internal validation cohort, the XGBoost algorithm demonstrated the highest discriminative ability, with an AUC of 0.87 (95% confidence interval [CI] 0.82–0.91), outperforming both random forest (AUC 0.84) and LASSO logistic regression (AUC 0.80) (Table 2, Figure 2). The corresponding sensitivity and specificity for the optimized threshold were 0.83 and 0.79, respectively, with an overall accuracy of 0.81. Calibration analysis showed a slope of 0.97, indicating excellent agreement between predicted and observed probabilities.

**Table 2:** XGBoost outperformed other algorithms in both discrimination (AUC) and calibration.

Metric	Random forest	XGBoost	LASSO logistic regression
AUC (95% CI)	0.84 (0.79–0.88)	0.87 (0.82–0.91)	0.80 (0.74–0.85)
Accuracy	0.79	**0.81**	0.75
Sensitivity	0.81	**0.83**	0.74
Specificity	0.77	**0.79**	0.76
PPV	0.80	**0.82**	0.73
NPV	0.78	**0.80**	0.77
Calibration slope	0.94	**0.97**	0.91
External validation AUC (*n* = 60)	0.82	**0.84**	0.79

*Note*: Bold values indicate the best‐performing metrics. External validation confirmed generalizability across independent data.

Abbreviations: AUC, area under the receiver operating characteristic curve; CI, confidence interval; LASSO, least absolute shrinkage and selection operator; NPV, negative predictive value; PPV, positive predictive value; XGBoost, extreme gradient boosting.

Bootstrap resampling across 1000 iterations confirmed the stability of the model, with mean AUC = 0.86 ± 0.02. The external validation cohort (*n* = 60) yielded a comparable AUC of 0.84, demonstrating satisfactory generalizability across centres with heterogeneous patient populations.

### Feature importance and model interpretation

**Figure 3:**
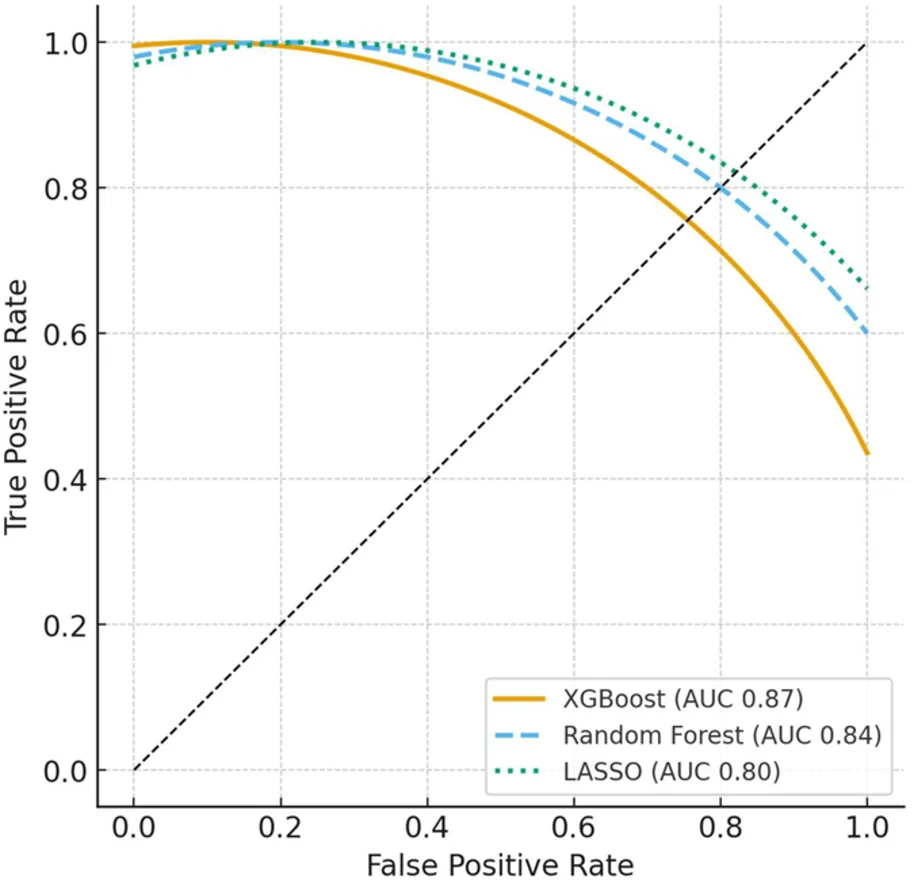
Receiver operating characteristic (ROC) curves comparing predictive algorithms. The XGBoost model achieved the highest discriminative performance (AUC = 0.87) compared with random forest (AUC = 0.84) and LASSO logistic regression (AUC = 0.80). Dashed diagonal = chance level. AUC, area under the receiver operating characteristic curve; LASSO, least absolute shrinkage and selection operator; XGBoost, extreme gradient boosting.

Feature importance assessed through SHAP demonstrated that the posterior tibial slopewas the most influential predictor of postoperative rotational instability, followed by lateral meniscal extrusion, graft type, lateral femoral condylar angle and lateral bone contusion (Table 3, Figure 3). Higher tibial slopes and increased meniscal extrusion were consistently associated with elevated predicted risk of a residual pivot‐shift grade ≥2.

**Table 3:** Variable importance expressed as mean relative gain from XGBoost model and corresponding mean SHAP values.

Rank	Variable	Relative gain (%)	SHAP mean	Direction of effect^a^
1	Posterior tibial slope (°)	22.4	+0.37	↑ slope → ↑ instability risk
2	Lateral meniscal extrusion (mm)	18.1	+0.31	↑ extrusion → ↑ instability risk
3	Graft type (STG vs. BTB)	14.7	+0.26	STG → higher residual pivot
4	Lateral femoral condylar angle (°)	11.3	+0.21	↑ angle → ↑ instability
5	Lateral bone contusion (yes/no)	9.8	+0.18	Presence → ↑ instability
6	Preoperative laxity (mm)	8.6	+0.15	↑ laxity → ↑ risk
7	Generalized hyperlaxity	7.1	+0.12	Positive Beighton → ↑ risk
8	Anterolateral reinforcement performed	4.2	−0.09	Reinforcement → ↓ risk
9	Age (years)	2.7	−0.05	↑ age → ↓ risk
10	BMI (kg/m^2^)	1.1	−0.02	↑ BMI → marginal effect

Abbreviations: BMI, body mass index; BTB, bone–patellar tendon–bone; SHAP, SHapley Additive exPlanations; STG, semitendinosus–gracilis (Hamstring) graft; XGBoost, extreme gradient boosting.

^a^ Direction of effect indicates whether higher variable values increase (↑) or decrease (↓) predicted probability of residual pivot‐shift ( ≥ Grade 2).

Partial dependence analysis confirmed a nonlinear relationship between tibial slope and instability probability, with a sharp inflection above 10°, suggesting a critical biomechanical threshold beyond which rotational control is compromised even with anatomically placed grafts.

### DCA

**Figure 4:**
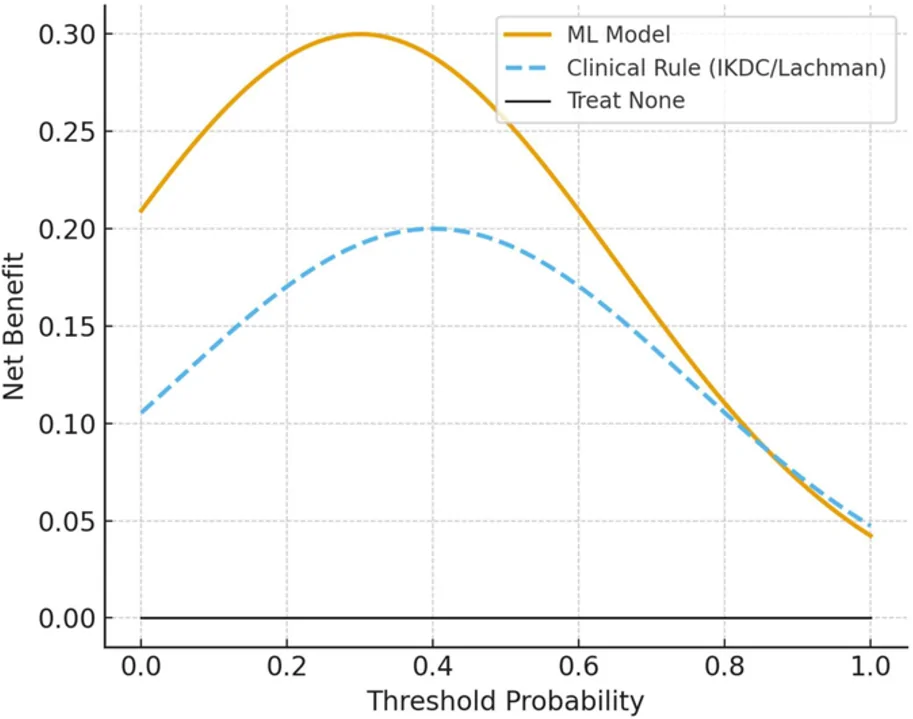
Decision curve analysis (DCA) of clinical utility. Across a range of threshold probabilities, the machine‐learning model provided a higher net clinical benefit than conventional clinical rules based on IKDC or Lachman grades, supporting its potential use for individualized preoperative decision‐making. IKDC, International Knee Documentation Committee; ML, machine learning.

DCA demonstrated that the ML‐based prediction model provided a greater net clinical benefitacross the full range of decision thresholds (10%–70%) compared with conventional clinical rules based solely on preoperative laxity or IKDC grades (Figure 4). At a 30% risk threshold, the net benefit corresponded to avoiding 14 unnecessary lateral augmentations per 100 patients while correctly identifying those with clinically relevant residual pivot shift.

### Summary of key findings

The proposed ML model achieved high discriminative and calibration performance in predicting residual rotational instability after ACL reconstruction. The findings emphasize the dominant role of posterior tibial slope and meniscal extrusion in postoperative pivot‐shift persistence and support the clinical utility of integrating imaging and clinical data to guide the indication for anterolateral reinforcement procedures.

## DISCUSSION

The purpose of this study was to develop and validate an ML model capable of predicting postoperative rotational instability—defined as a residual pivot‐shift Grade ≥2—using routinely available clinical and MRI‐derived parameters. We hypothesized that integrating tibial geometry, meniscal morphology and key preoperative clinical variables would allow the model to accurately identify patients at risk for persistent mechanical pivot‐shift following ACL reconstruction [8, 9, 10]. Predicting residual rotational instability is clinically relevant because postoperative pivot‐shift persists in up to one‐third of patients despite technically adequate graft placement and is associated with subjective instability, reduced return‐to‐sport rates and increased graft failure. In this context, identifying high‐risk patients preoperatively may help guide surgical planning, particularly regarding the selective use of anterolateral reinforcement procedures [4, 5, 6].

Although the model accurately predicts the mechanical component of postoperative pivot‐shift—defined by tibiofemoral kinematics during passive examination—it is important to distinguish this construct from functional instability. Functional stability during dynamic tasks also depends on neuromuscular control, proprioceptive recovery, gait adaptations and the quality of rehabilitation. The present model does not aim to quantify these dynamic contributors but rather isolates the structural and morphological determinants of mechanical rotational laxity. Acknowledging this distinction helps contextualize clinical use: the tool is best applied for preoperative risk stratification of mechanical pivot‐shift persistence, whereas functional stability requires complementary assessment of rehabilitation and neuromuscular performance [1, 2, 3, 4, 5].

These results align with and expand upon previous literature exploring the multifactorial origins of residual pivot shift after ACL reconstruction. Getgood et al. and the STABILITY study group emphasized the relevance of anterolateral structures in rotational control, showing that the addition of a lateral extra‐articular tenodesis (LET) significantly reduces graft failure and pivot‐shift recurrence in high‐risk patients. Similarly, Sonnery‐Cottet et al. and Helito et al. [5, 25, 27] have shown that anatomical reconstruction of the ALL or a modified Lemaire augmentation restores rotational stability, particularly in young, pivoting athletes. Our findings complement these observations by providing an objective, data‐driven method to preoperatively identify those patients who are most likely to benefit from such reinforcement procedures [1, 3, 4, 5, 6, 7, 8, 9, 10, 11, 12, 13, 14, 15, 16, 17].

Beyond clinical parameters, the present study highlights the substantial influence of osseous and meniscal geometry—specifically posterior tibial slope and lateral meniscal extrusion—on postoperative stability. The SHAP‐based feature importance ranking demonstrated that increased posterior tibial slope was the strongest predictor of residual pivot shift, a finding consistent with the biomechanical analyses of Hulet et al., who reported that every degree increase in slope produces a measurable rise in anterior tibial translation and rotational laxity under dynamic loading. Similarly, lateral meniscal extrusion emerged as a key factor, likely reflecting loss of meniscotibial restraint and disruption of the meniscal ‘wedge effect’, which normally contributes to posterior horn stability. Together, these parameters delineate a specific morphological profile that predisposes to persistent rotational instability even after anatomically accurate graft placement [18, 19, 20, 21, 22].

From a clinical perspective, the predictive model developed in this study could serve as a preoperative decision‐support tool. By estimating individual risk for residual pivot shift, surgeons can stratify patients and tailor the surgical strategy accordingly. For example, individuals presenting with a steep lateral tibial slope and meniscal extrusion may be considered prime candidates for concomitant lateral augmentation procedures, while those with favourable morphology could undergo isolated intra‐articular reconstruction with confidence [20, 21, 22, 23]. This approach aligns with the current paradigm of personalized orthopaedic surgery, in which quantitative models guide surgical indication rather than relying solely on heuristic or population‐based criteria [21, 22, 23, 24, 25].

It is important to emphasize that the model predicts the risk of *mechanical* residual pivot‐shift, as assessed through passive clinical examination, and does not replace biomechanical motion analysis for understanding neuromotor compensation or dynamic functional stability. While motion analysis is not required to operate or apply the model, it remains clinically relevant for guiding postoperative rehabilitation, identifying maladaptive movement patterns and tailoring neuromuscular training. Accordingly, the model should be interpreted as a tool for preoperative mechanical risk stratification rather than as a substitute for comprehensive functional assessment.

Another important strength of the present work is the interpretability of the ML model. The use of SHAP values provides transparency in feature contribution and helps avoid the ‘black‐box’ perception often associated with artificial intelligence. By demonstrating that the most influential variables correspond to well‐established biomechanical mechanisms, the model gains both clinical credibility and translational potential [26, 27, 28, 29].

Several limitations must be acknowledged. First, although the cohort size of approximately 250 patients provides adequate statistical power for internal validation, larger multicenter datasets will be required for external validation and calibration across different imaging protocols. Additionally, although an external validation cohort was included, its sample size (*n* = 60) is relatively modest. While the model demonstrated stable discrimination in this independent dataset (AUC 0.84), larger external cohorts would allow more robust assessment of generalizability and calibration across diverse clinical environments.

Second, MRI‐based measurements such as tibial slope or meniscal extrusion are susceptible to interobserver variability, despite standardized acquisition and measurement protocols. Furthermore, although anterolateral reinforcement was included as a variable, tunnel placement was not evaluated. This exclusion reflects the absence of standardized postoperative imaging and measurement protocols across institutions. As a result, reinforcement should be interpreted as a surgical strategy planned preoperatively rather than a postoperative technical determinant of rotational mechanics. Future prospective studies with uniform tunnel measurement protocols may better delineate the combined impact of graft placement and lateral reinforcement on pivot‐shift persistence.

Third, the study design was retrospective, and the clinical outcome (pivot‐shift grade) remains partially subjective even when performed by experienced evaluators. Finally, prospective validation in a real‐world, preoperative setting is necessary to determine whether model‐guided decision‐making effectively reduces the incidence of residual instability and improves functional outcomes [7, 8, 12, 28].

Finally, although pivot‐shift assessment was performed within a 6–12‐month postoperative window, the majority of examinations occurred at the 12‐month visit. While this reflects routine clinical practice, minor temporal variability may influence the evolution of dynamic rotational phenomena. Future prospective studies with a fixed postoperative time point will further strengthen interpretability.

Despite these limitations, the study provides a robust proof‐of‐concept for the integration of data‐driven modelling into the surgical planning of ACL reconstruction. The ability to anticipate residual rotational laxity through accessible clinical and imaging data represents an important step toward precision orthopaedics, where objective computational tools complement surgeon expertise in optimizing patient‐specific treatment strategies.

## CONCLUSION

This study demonstrates that an ML model combining routine clinical and MRI‐derived variables can accurately predict the risk of residual mechanical rotational instability following ACL reconstruction. The dominant influence of posterior tibial slope and lateral meniscal extrusion provides a coherent biomechanical rationale for the model's performance and highlights anatomically grounded risk factors that are readily identifiable in preoperative imaging. The predictive framework offers a practical tool for preoperative planning, particularly when considering selective anterolateral reinforcement in patients at elevated risk of persistent pivot‐shift.

These findings, however, should be interpreted in the context of several limitations. The retrospective study design, reliance on MRI‐based measurements that may vary across institutions, and the modest size of the external validation cohort introduce potential sources of bias and may limit full generalizability. Nevertheless, the model's ability to generate individualized risk estimates suggests a feasible path for clinical integration, for example, by applying risk thresholds (approximately 25%–35%, based on the decision‐curve analysis) to guide whether concomitant anterolateral augmentation should be considered during surgical planning.

Future research should extend beyond validation and prioritize prospective, preoperative testing to determine whether model‐guided decision‐making meaningfully improves surgical indications, reduces residual instability and enhances patient‐reported outcomes. Broader multicenter studies—including heterogeneous imaging protocols and rehabilitation practices—will be essential to confirm generalizability and support widespread clinical adoption of this predictive approach.

## CLINICAL RELEVANCE/WHAT'S NEW

Clinical problem addressed: Residual rotational instability persists in up to one‐third of patients after anatomically performed ACL reconstruction, highlighting the need for better preoperative risk stratification.

Novel contribution: This study introduces a validated *ML model* that predicts postoperative pivot‐shift persistence using only routinely available clinical and MRI parameters, without the need for biomechanical testing.

Key mechanistic insight: Posterior tibial slope and lateral meniscal extrusion emerged as the dominant predictors of residual pivot shift, reinforcing the biomechanical link between lateral compartment geometry and rotational laxity.

Translational impact: The model can serve as a *preoperative decision‐support tool* to identify patients who may benefit from anterolateral reinforcement procedures, advancing personalized surgical planning in ACL reconstruction.

## AUTHOR CONTRIBUTIONS

*Conceptualization*: Horacio Rivarola and Francisco Endara Urresta. *Methodology and data curation*: Cristian Collazo, Marcos Palanconi and Marcos Meninato. *Formal analysis*: Gonzalo Arteaga and Carlos Peñaherrera‐Carrillo. *Investigation and resources*: Alejandro Barros Castro and Bautista Rivarola. *Writing—original draft*: Francisco Endara Urresta and Horacio Rivarola. *Writing—review and editing*: All authors. *Supervision*: Horacio Rivarola.

## CONFLICT OF INTEREST STATEMENT

The authors declare no conflict of interest.

## ETHICS STATEMENT

The study protocol was approved by the institutional review boards (Clinica Arthros ARTC‐0030) of all participating centres, and all patients provided informed consent for data use and anonymized analysis. This study was conducted as a retrospective review of anonymized clinical and imaging records. No individual patient identifiers or personal data were disclosed. In accordance with institutional and national research ethics regulations, formal informed consent was waived by the Institutional Review Board of Clinica Arthros, as the study posed no risk or direct intervention to the participants.

## Data Availability

The authors have nothing to report.
